# Mu opioid receptor availability in people with psychiatric disorders who died by suicide: a case control study

**DOI:** 10.1186/1471-244X-12-126

**Published:** 2012-08-28

**Authors:** Elizabeth Scarr, Tammie Terese Money, Geoffrey Pavey, Jaclyn Neo, Brian Dean

**Affiliations:** 1 Molecular Psychiatry Laboratory, Florey Institute of Neuroscience and Mental Health, Melbourne Brain Centre, University of Melbourne, Parkville, VIC 3010, Australia; 2 Department of Psychiatry, Melbourne Brain Centre, The University of Melbourne, Parkville, VIC 3010, Australia

## Abstract

**Background:**

Mu opioid receptors have previously been shown to be altered in people with affective disorders who died as a result of suicide. We wished to determine whether these changes were more widespread and independent of psychiatric diagnoses.

**Methods:**

Mu receptor levels were determined using [^3^ H]DAMGO binding in BA24 from 51 control subjects; 38 people with schizophrenia (12 suicides); 20 people with major depressive disorder (15 suicides); 13 people with bipolar disorder (5 suicides) and 9 people who had no history of psychiatric disorders but who died as a result of suicide. Mu receptor levels were further determined in BA9 and caudate-putamen from 38 people with schizophrenia and 20 control subjects using [^3^ H]DAMGO binding and, in all three regions, using Western blots. Data was analysed using one-way ANOVAs with Bonferroni’s Multiple Comparison Test or, where data either didn’t approximate to a binomial distribution or the sample size was too small to determine distribution, a Kruskal-Wallis test with Dunn’s Multiple Comparison Test.

**Results:**

[^3^ H]DAMGO binding density was lower in people who had died as a result of suicide (p<0.01). People with schizophrenia who had died as a result of suicide had lower binding than control subjects (p<0.001), whilst people with bipolar disorder (non- suicide) had higher levels of binding (p<0.05). [^3^ H]DAMGO binding densities, but not mu protein levels, were significantly decreased in BA9 from people with schizophrenia who died as a result of suicide (p<0.01).

**Conclusions:**

Overall these data suggest that mu opioid receptor availability is decreased in the brains of people with schizophrenia who died as a result of suicide, which would be consistent with increased levels of endogenous ligands occupying these receptors.

## Background

Although the dynamics that contribute to a person dying by suicide are multifactorial, highly complex and vary between individuals [1], studies consistently suggest that psychiatric disorders confer an increased risk of suicide. For example, a Danish study found that nearly 50% of people who died as a result of suicide either had been or were psychiatric inpatients [2]. A retrospective study in Hong Kong discovered that 80% of the people who died by suicide had a psychiatric disorder, with 29% of these having a co-morbid diagnosis [3]. This study also showed the highest risk of suicide was in people with mood disorders (50%), with suicides in psychotic disorders showing a gender difference (females: 35.1%; males: 19.8%). Most significantly, the study showed that a psychiatric diagnosis had an adjusted odds ratio of 28.67 for risk of suicide.

The concept that psychiatric disorders significantly increase the risk of suicide is highlighted by a systematic literature review which showed that, of all factors studied, psychiatric disorders had the strongest association with suicide (all psychiatric disorders 91%; of which mood disorders accounted for 55% and co-morbid diagnoses 44%) [4]. A recent meta-analysis, which used data from both case–control studies and cohort studies, reported that the relative risk of suicide that could be attributed to any psychiatric disorder was 7.5% for males and 11.7% for females [5]. Together, these data demonstrate that there is an urgent need to understand the drives to suicidal tendencies in people with psychiatric illnesses; the ultimate goals of such investigations being to identify markers for such tendencies and/or to develop interventions to reduce this behaviour.

There is now a body of literature which supports the hypothesis that neurochemical changes in the brain are associated with suicidal behaviour, which include roles for the central serotonergic system [6], the signal transducer protein kinase C [7] and the glutamatergic system [8]. There are also studies linking the endogenous opioidergic system to the major psychiatric disorders [9,10] and suicide [11]. In the case of the mu opioid receptor (OPRM), it has been reported that these receptors are increased in the inferior frontal, cingulate, postcentral, medial temporal and lateral occipitotemporal gyri as well as the thalamus [12] of young (<41 years) people who had died as a result of suicide. OPRM were also reported to be increased in the frontal cortex and caudate [13] from people who had died as a result of suicide compared to those from age and sex matched control subjects. Significantly, one of these studies [12] only included people with depression among those who had died by suicide, whilst the second study predominantly consisted of people with undetermined diagnoses or depression (one subject had schizophrenia) [13]. Thus, it is not clear if there is an interaction between psychiatric disorder and OPRM in people who died by suicide. Finally, in a study that did not assign psychiatric diagnoses to the cohorts, it has been reported that OPRM in the prefrontal cortex from people who died by suicide bound agonists with a higher affinity than that for people who did not die by suicide. However, a decreased binding affinity was reported for the pre- and post-central gyri from people who died as a result of suicide [14].

Given the potential for changes in OPRM to be important in the predisposition of an individual to attempt suicide and the relative paucity of studies on OPRM, we decided to measure the density of OPRM using [^3^ H][D-Ala^2^, N-MePhe^4^, Gly-ol]-enkephalin (DAMGO) binding in BA24 from people with no history of psychiatric disorders (controls), people who had died by suicide and had no discernable history of psychiatric disorders (suicides) and people with schizophrenia, major depressive disorder (MDD) or bipolar disorder (BP); some of whom had died by suicide.

Following our initial studies we went on to measure [^3^ H]DAMGO binding in BA 9 and the caudate-putamen (CPu) from people with schizophrenia who had or had not died as a result of suicide and compared these data to those from age/sex matched controls (non-suicides) to begin to determine the extent of altered OPRM binding in these people. Finally, to determine if changes in radioligand binding were associated with changes in levels of OPRM protein, we used Western blotting and an anti-human OPRM antibody to measure protein levels in BA9, 24 and the caudate from the schizophrenia study cohorts.

## Methods

Approval for this study was obtained from the Ethics Committee of the Victorian Institute of Forensic Medicine and the Mental Health Research and Ethics Committee of Melbourne Health (MHREC 2001.613). In all cases, tissue was obtained, post-mortem, after receiving written consent from the closest identifiable next-of kin. In compliance with State laws of the day, the source of the tissue collected up to 1999 was de-identified on completion of the case history review, tissue collected after 2002 was not de-identified. Tissues were provided by the Victorian Brain Bank Network as coded samples so that investigators cannot identify the donor.

### Diagnostic evaluation

Case history reviews were conducted using the Diagnostic Instrument for Brain Studies (DIBS) [15,16], with a psychologist and psychiatrist reaching a diagnostic consensus using DSM-IV criteria [17]. The inter-rater reliability of this instrument is 94% (mean kappa = 0.88; 95% confidence interval = 0.7 – 1.0) [18]. Following the review, duration of illness (DOI: time from first contact with psychiatric services to death) was calculated and latest prescribed doses of psychoactive drugs converted to standardised drug doses [19] ( Additional file 1: Table S1). Importantly, CNS tissue was collected from cadavers refrigerated within 5 hours of being found, this has been shown to markedly slow autolysis processes and protein degradation [20]. The average time from discovery to autopsy was approximately 36 hours. To ensure preservation of the CNS molecular cytoarchitecture, samples were processed using a standardised procedure and rapidly frozen to −70°C after removal at autopsy [21]. In cases where death was witnessed, the post-mortem interval (PMI) was the time between death and autopsy. Where death was not witnessed, PMI was calculated as the interval mid-way between the donor last being seen alive and being found dead. Central pH was determined as described previously [22]; this being recognised as a good indicator of tissue and protein preservation [23].

### Cohort selection and tissue processing

Blocks of BA24 (ventral anterior cingulate gyrus, around the genu of the corpus callosum) were removed from the brains of 51 controls, 38 people with a diagnosis of schizophrenia (12 suicides), 20 people with a diagnosis of MDD (15 suicides), 13 people with a diagnosis of BP (5 suicides) and 9 suicides with no discernible history of a psychiatric disorder (see Table 1 for cohort summary and Additional file 1: Table S1 for details).

**Table 1:** A summary of the demographic and collection data (mean ± SEM) for the cohort used in the determination of markers for mu opioid receptors

**Group**	**Sample size**	**Age (years)**	**PMI (hours)**	**Brain pH**
Controls	51	52.90 ± 2.62	43.73 ± 2.34	6.280 ± 0.036
Suicides	9	44.56 ± 5.93	33.69 ± 3.67	6.427 ± 0.066
MDD	5	65.00 ± 8.79	34.70 ± 4.34	6.266 ± 0.103
MDD (suicide)	15	50.93 ± 4.69	39.45 ± 3.69	6.572 ± 0.053
BP	8	67.25 ± 2.827	36.03 ± 6.688	6.269 ± 0.072
BP (suicide)	5	41.40 ± 4.214	32.10 ± 4.173	6.348 ± 0.074
Schizophrenia	26	54.38 ± 2.619	38.07 ± 2.395	6.190 ± 0.052
Schizophrenia (suicide)	12	29.25 ± 2.769	44.67 ± 4.030	6.261 ± 0.043

Blocks of BA9 (lateral surface of the frontal lobe, including the middle frontal gyrus superior to the inferior frontal sulcus) and the CPu were excised from the brains of the 36 people (11 suicides) with schizophrenia (tissue unavailable from 2 people) and 20 control age and sex subjects.

The radioligand binding assays were conducted as single point saturation studies [24], which gives a good estimate of total available binding sites. Importantly, the concentration of radioligand used was sufficiently high to overcome the minor changes in radioligand binding affinity that occurs in human CNS tissue [25] ensuring this approach gives a good estimation of total binding sites within the tissue [26]. Thus, 5x 20 μm sections (3 total binding; TB, 2 non-specific binding; NS) were cut from each tissue block using a cryomicrotome (CM1800, Leica Microsystems) and thaw mounted onto gelatinised microscope slides. Approximately 100 mg of gray matter for use in Western blots was excised from the slices these sections were taken from and stored at −80°C.

### Radioligand binding

^3^ H]DAMGO binding (3.3 x10^-9^ M) was measured in the presence (NS) and absence (TB) of 10^-6^ M naloxone; adapted from previous studies [12,27]. Sections were incubated in 50 x10^-3^ M Tris–HCl, pH7.4, for 1 hour. After incubation, sections were washed in ice-cold assay buffer, dipped in ice-cold water and thoroughly dried prior to being desiccated overnight in paraformaldehyde fumes. The sections, alongside ^3^ H]micro-scales™, were apposed to BAS-TR2025 imaging plates (14 days), which were scanned using a BAS5000 high resolution phosphoimager. The phosphoimage intensities were measured by comparison to the intensity of segments on ^3^ H]microscales™ using AIS image analysis software. Results were expressed as specific binding (TB minus NS) in dpm/mg estimated wet weight tissue equivalents (ETE) and converted to fmol/mg ETE [26].

### Western blots

Homogenates were prepared from CNS tissue, at 5% w/v, in 10 x10^-3^ M Tris, (pH 7.4), containing 1% sodium dodecylsulphate and 1 x10^-3^ M fresh sodium orthovanadate and diluted in reducing buffer to give final protein concentrations of 1.0 mg/ml. Optimisation experiments showed that different protein concentrations from each region were within the linear range of the detection system. Thus the total protein loaded was; BA 9: 30 μg, BA 24: 10 μg and caudate: 40 μg. The proteins in each sample (run in duplicate) were separated using polyacrylamide gel electrophoresis (4% stacking gel, 10% running gel). They were transferred to nitrocellulose membranes overnight in Towbin transfer buffer [28]. Equal protein loading and transfer was confirmed by staining with 0.1% ponceau S in 3% trichloroacetic acid. Membranes were blocked for an hour at room temperature in Tris buffered saline with 1% Tween 20 (TTBS) and incubated with rabbit anti-OPRM antibody (1/2000 in TTBS) overnight at 4°C. This was followed by incubation with goat anti-rabbit antibody conjugated to horseradish peroxidise (1/2000 in TTBS) for an hour at room temperature. The antigenic reaction was visualised using enhanced chemiluminescence (ECL) and imaged using a Kodak Image Station 440CF. The sum intensity of the antigenic bands was measured using Kodak 1D software.

To control for inter-blot variability, an internal control (IC) sample [29], was run (in duplicate) on every gel, which were imaged so that the sum intensity of the IC fell within a previously established range. The sum intensities of all samples were standardised as a ratio of Internal Control (ratio IC).

### Statistics

All data sets were analysed using the D’Agostino & Pearson omnibus normality test to determine whether they approximated to Gaussian distribution. If the data was normally distributed, a Grubb’s test was used to determine the presence of outliers. Due to small sample sizes, age, PMI and brain pH for the groups were compared using a Kruskal-Wallis test with Dunn’s Multiple Comparison Test. Pearson product–moment correlations, assuming a straight line fit, were used to determine if there was a relationship between these factors and experimental data. Interpretation of Pearson product–moment correlations was assisted by published guidelines [30]; with an r value of 0.3 (r^2^ = 0.09) deemed to describe a typical relationship and a value of 0.5 (r^2^ = 0.25) describing a substantial relationship.

Where an association was found, the impact of the factors on the experimental data was determined by analysis of covariance (ANCOVA). For the radioligand binding data, a Student’s unpaired *t*-test was used to determine whether there were changes in binding density with suicide. Due to the small samples sizes for some groups, a Kruskal-Wallis test with Dunn’s Multiple Comparison Test, or a general linear model (GLM) which is most suitable for dealing with complex data sets were employed to identify variations in binding density between groups. Finally, in the schizophrenia/control cohort, one-way ANOVAs with Bonferroni’s Multiple Comparison Test were used to identify variations in [^3^ H]DAMGO binding and OPRM protein levels between groups in each CNS region. GLMs and ANCOVAs were conducted using Minitab (Release 13.31, http://www.minitab.com); all other statistical analyses were carried out using Graphpad Prism (version 5.04 for Windows, Graphpad Software, San Diego, California, USA).

## Results

### Demographic and tissue collection data

The final recorded antipsychotic dose (chlorpromazine equivalents) and duration of illness in the people with schizophrenia were the only collection-related variables not normally distributed. There were significant variances associated with age (Kruskal-Wallis statistic = 30.80, p<0.0001) and pH (Kruskal-Wallis statistic = 27.87, p<0.001), but not PMI (Kruskal-Wallis statistic = 9.24, p = 0.24) between the groups. Post-hoc Dunns Multiple Comparison test revealed that the variance with age was due to the schizophrenia _ suicide group being younger than the control (p<0.01), MDD_non_suicide (p<0.05), BP_non_suicide (p<0.001) and the schizophrenia_non_suicide groups (p<0.01; Additional file 2: Table S2). Using the same approach, the variance with brain pH was found to be due to the tissue from the MDD_suicide group being higher than for the control (p<0.01) and schizophrenia_suicide (p<0.01) and schizophrenia_non_suicide (p<0.001) groups ( Additional file 2: Table S2).

### [^3^ H]DAMGO Binding in BA24 and suicide

**Figure 1:**
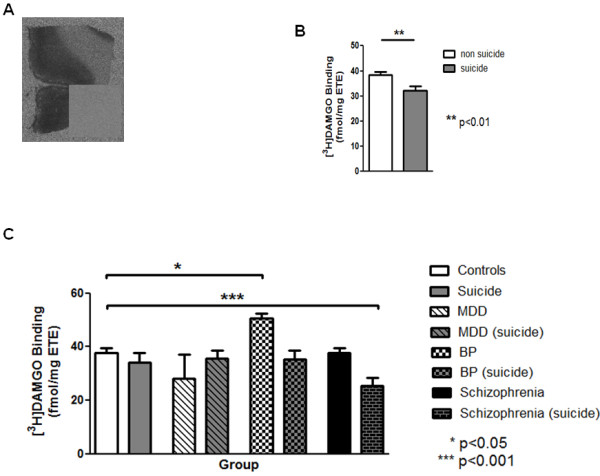
**a) Representative autoradiograph of total [**^**3**^ **H]DAMGO binding in BA24.** Insert shows non-specific binding. Results for the radioligand are represented graphically (mean ± SEM, fmole/mg ETE): **b**) Effect of suicide *per se* on levels of [^3^ H]DAMGO binding in BA24. **c**) Effect of psychiatric disorder and suicide on levels of [^3^ H]DAMGO binding in BA24.

[^3^ H]DAMGO binding was homogenous across BA24 (see Figure 1a) therefore an integrated measure was taken across all cortical laminae. [^3^ H]DAMGO binding from people who had or had not died from suicide approximated to a Gaussian distribution and a Grubbs test revealed no outlying data. Significantly, there was a 16% decrease in the density of radioligand binding to OPRM in BA24 from people who had died by suicide (n = 41) compared to those who had not died by suicide (n = 81, p = 0.008, see Table 2 and Figure 1b).

**Table 2:** Levels of [3 H]DAMGO (mean ± SEM) binding in subjects with schizophrenia, major depressive disorder, bipolar disorder or no history of psychiatric disorder who did or did not die as a result of suicide

**Group BA 24**	**Sample size**	**[3 H]DAMGO binding (fmole/mg ETE)**	**Analysis parameters**	**P value**
Suicide	41	32.25 ± 1.76	t = 2.7, df = 120 (non-suicide)	0.008
Non-suicide	81	38.34 ± 1.34		
Diagnosis/suicide	BA 24			
Controls	51	37.63 ± 1.83		
Suicides	9	34.02 ± 3.80	t = 0.88 (controls)	n.s
MDD	5	27.95 ± 9.06	t = 1.84 (controls)	n.s
MDD (suicide)	15	35.69 ± 2.84	t = 0.58 (controls)	n.s
BP	8	50.51 ± 1.98	t = 3.00 (controls)	n.s
BP (suicide)	5	35.38 ± 3.07	t = 0.43 (controls)	n.s
Schizophrenia	26	37.80 ± 1.83	t = 0.04 (controls)	n.s
Schizophrenia (suicide)	12	25.31 ± 3.17	t = 3.39 (controls) t = 3.21 (schizophrenia)	<0.05
Schizophrenia/suicide	BA 9			
Controls	20	31.65 ± 2.11		
Schizophrenia	26	34.40 ± 1.32	t = 1.15 (controls)	n.s
Schizophrenia (suicide)	12	24.12 ± 2.27	t = 2.57 (controls) t = 3.68 (schizophrenia)	<0.05 <0.05
	CPu			
Controls	20	25.40 ± 2.16		
Schizophrenia	24	23.72 ± 1.37	t = 0.67 (controls)	n.s
Schizophrenia (suicide)	11	16.29 ± 2.42	t = 2.97 (controls) t = 2.50 (schizophrenia)	<0.05 <0.05

The group comparison the analysis factors refer to is given in brackets below the parameters. Abbreviations: *n.s*. = not significant.

#### [^3^ H]DAMGO Binding in BA24, suicide and psychiatric diagnoses

To determine if the changes in OPRM in BA24 showed any suicide x psychiatric diagnosis, we divided the data according to suicide status within each clinical diagnoses. This showed changes in [^3^ H]DAMGO binding varied with suicide status (F_7,114_ = 4.16, p = 0.0004) and was due to a 34% increase in binding in the BP_non_suicide group and a 33% decrease in the schizophrenia _ suicide group (see Table 2 and Figure 1c).

### Potential confounds

There were substantial correlations between [^3^ H]DAMGO binding and age in controls, MDD_non_suicide, MDD_suicide, BP_non_suicide and schizophrenia_suicide as well as between binding and pH in MDD and BP_suicide ( Additional file 2: Table S2). Furthermore, when [^3^ H]DAMGO binding was analysed with age and pH as cofactors, age (F_1,89_ = 23.12, p<0.0001) and pH (F_1,89_ = 3.91, p = 0.05) were shown to contribute to the variance associated with the binding data. However, even taking these factors into account, the suicide x psychiatric diagnoses differences were still strongly significant (F_7,89_ = 2.93, p = 0.008).

### Mu opioid receptors and suicide in schizophrenia

#### [^3^ H]DAMGO binding

**Figure 2:**
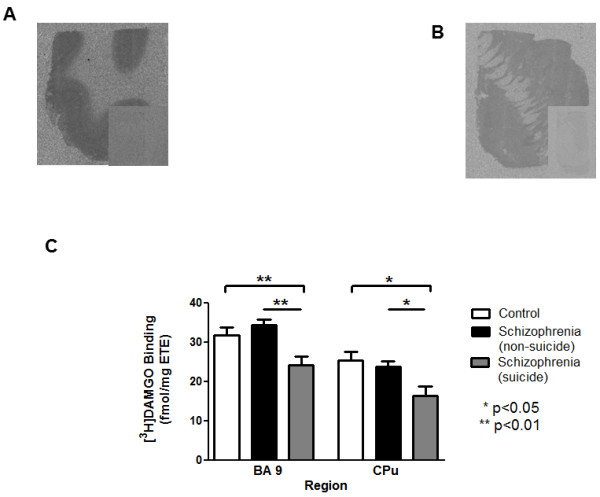
**Representative autoradiographs of total [**^**3**^ **H]DAMGO binding in a) BA9 and b) caudate-putamen; inserts show non-specific binding. c**) Effect of suicide on radioligand binding in these areas are represented graphically (mean ± SEM, fmole/mg ETE).

Whilst the primary focus of this study was the potential interactions between diagnoses x suicide, a sub-analyses of the BA 24 data revealed that there was a 33% decrease in binding in the schizophrenia_suicide group compared to the schizophrenia_non_suicide group (Table 2), prompting further study of the potential interactions between suicide x schizophrenia in other CNS regions. Subsequently, we measured [^3^ H]DAMGO binding in BA9 (Figure 2a) and CPu (Figure 2b) from people with schizophrenia_ suicide, schizophrenia_non_suicide and age matched controls only. These data were shown to approximate to a Gaussian distribution in both regions and Grubbs test revealed one outlier in the data in the CPu from people with schizophrenia (Z >2.82). For transparency, analyses are presented with and without that outlier. With the outlier, there was a significant variation in [^3^ H]DAMGO binding in the CPu with diagnoses (With outlier: F_2,53_ = 3.90, p = 0.026; without outlier: F_2,52_ = 4.66, p = 0.013). Binding in the schizophrenia _ suicide group was decreased by 36% compared to controls and 31% compared to schizophrenia_non_suicide (Table 2, Figure 2c).

[^3^ H]DAMGO binding also varied with diagnoses in BA9 (F_2,55_ = 6.81, p = 0.0023), due to a 24% decrease in binding in the schizophrenia_suicide group compared to controls and 30% compared to schizophrenia_non_suicide (Table 2, Figure 2c). In neither region did [^3^ H]DAMGO binding in the schizophrenia_non_suicide differ from that in controls.

### Potential confounds

Pearson product–moment correlations indicated that age, pH and DOI but not PMI or chlorpromazine equivalents, had significant associations with [^3^ H]DAMGO binding ( Additional file 2: Table S2). Significantly, reanalysing the schizophrenia cohort data with age and pH as covariates showed that although age (F_1,161_ = 25.48, p<0.0001) and pH (F_1,161_ = 5.64, p = 0.019) contributed to the variance in binding, the variation associated with suicide was still significant (F_2,161_ = 3.33, p = 0.038).

### OPRM protein

**Figure 3:**
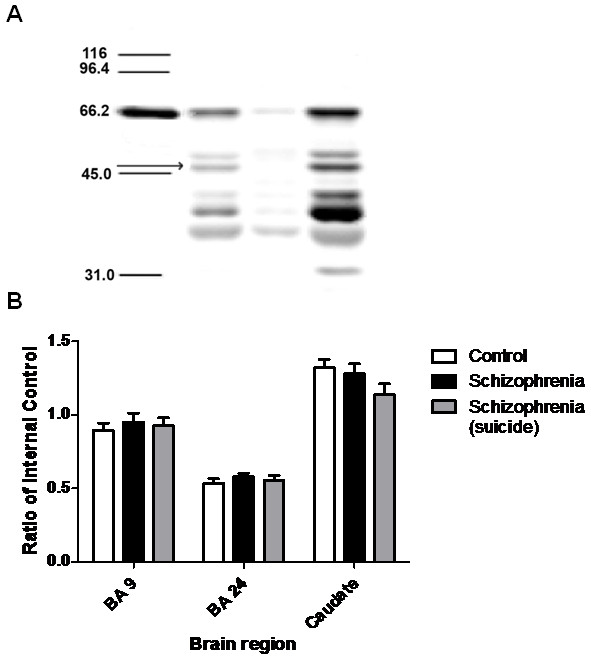
**a) Western blot showing the levels of OPRM in (left to right): Molecular weight standards in lane 1, values given in kDa; membrane, nuclear and cytosol fractions, in lanes 2–4 respectively.** Band measured in subsequent experiments identified by the arrow. **b**) Levels of OPRM protein in BA24, 9 and CPu from controls and people with schizophrenia who did or did not die as a result of suicide are represented graphically (mean ± SEM, ratio of internal control).

The antigenic band measured in these studies had the predicted molecular weight for MOR and, in our hands, showed the expected rank order of intensity (reflecting OPRM levels) across different brain regions (i.e. thalamus > cortex > caudate; data not shown) [12,27]) and was present in thalamic membrane and cytosolic [31-33] but not nuclear fractions (Figure 3a). 

OPRM data showed a Gaussian distribution with no outliers. There was no variance in OPRM protein levels between diagnostic groups in any region (BA9: F_2,55_ = 0.644, p = 0.53; BA24: F_2,55_ = 0.953, p = 0.392; caudate: F_2,53_ = 1.22, p = 0.303; see Figure 3b).

Overall, there were no correlations between age, pH, DOI or chlorpromazine equivalents and OPRM levels ( Additional file 2: Table S2). However, there were substantive relationships between OPRM level and age (r^2^ = 0.4809) and pH (r^2^ = 0.3758) in BA9 from the schizophrenia_ suicide group. An ANCOVA revealed that neither age (F_1,53_ = 0.67, p = 0.417) nor pH (F_1,53_ = 2.12, p = 0.151) had a significant effect on OPRM levels in BA 9. Furthermore, taking these 2 factors into account did not affect the outcome of the analysis (F_2,53_ = 0.72, p = 0.49).

## Discussion

This study has shown decreased ^3^ H]DAMGO binding in BA24, BA9 and the CPu from people with schizophrenia who died by suicide which, in BA24, was first revealed as a significant decrease in ^3^ H]DAMGO binding in larger cohort where people who had died by suicide were compared to those who had not. Significantly there were no differences in ^3^ H]DAMGO binding in BA 24 from people with either MDD or BP who had died as a result of suicide, making this a specific association with suicide in schizophrenia. However, this study did show an increase in ^3^ H]DAMGO binding in BA24 from people with BP who did not died by suicide. Some caution is needed in the interpretation of this finding in BP because of the relatively small number of individuals making up this cohort, particularly given that lithium treatment has been shown to increase OPRM expression in rats [34]. Although this novel finding deserves further investigation, it was not pursued in this study as it was not central to investigating the interactions between suicide x psychiatric disease.

Our study showed that ^3^ H]DAMGO binding increased with age, replicating previous studies that have reported this relationship [12,13]. Significantly, although DAMGO binds to all OPRM splice variants with similar affinities [35] our data showed that levels of OPRM protein did not vary with age. Thus, our data is the first to indicate that the number of available ^3^ H]DAMGO binding sites increases with age in the absence of changes in levels of OPRM, which would be consistent with decreasing levels of endogenous ligand occupancy with age. This hypothesis is supported from previous data on endogenous opioids in rat CNS [36] and in pain responsiveness in humans [37]. Together, these data suggest levels of endogenous opioid ligands decrease with age.

We have shown that decreased ^3^ H]DAMGO binding is detectable across several CNS regions in people with schizophrenia who died as a result of suicide, even when the effects of confounding factors such as age and tissue pH are taken into account, with no difference in binding levels between controls and people with schizophrenia. Our data partly replicates a previous study that showed no changes in binding to OPRM in people with schizophrenia who “had died from….. natural causes” [38] but contrasts with another report showing decreased binding to OPRM in the caudate from people with the disorder [39]. The latter study used tissue from people who had died as a result of either suicide or pneumonia but did not explore whether there was an association between low levels of binding and suicide; thus, it is possible that the decrease reported was due to suicide. However, our finding of low ^3^ H]DAMGO binding density in suicide *per se* does not agree with previous studies which report increased OPRM binding in the inferior frontal, cingulate, postcentral, medial temporal and lateral occiptotemporal gyri as well as the thalamus of young suicides [12] and in the frontal cortex and caudate [13] from people who had died by suicide or unaltered levels of radioligand binding in prefrontal cortex and prepost central gyri from people who suicided [14]. Notably, in the earliest studies investigating suicide and OPRM, tissue was obtained from individuals who had died by suicide and predominantly had MDD or BP; thus, these data are in contrast with our findings of no difference in levels of OPRM binding in people with MDD or BP who had died by suicide. By contrast, our finding of no change in ^3^ H]DAMGO binding in people with mood disorders or no history of psychiatric illness who had died by suicide is consistent with a previous study [14], which did not report on psychiatric diagnoses.

As stated earlier, our findings of decreased ^3^ H]DAMGO binding with no changes in OPRM protein would be consistent with a change in receptor availability rather than receptor number. There are three scenarios that would account for this decrease in agonist binding without a change in overall levels of protein. Firstly, it is possible that there is a change in the confirmation of the receptor which affects the binding of the ligand. Secondly, the biochemical modifications that are required for the insertion of the receptor into the membrane could be aberrant, reducing the availability of the receptor but not affecting the overall number of receptors. Finally, the simplest explanation is that the decrease in binding is due to competition for the binding site between the radioligand and an endogenous ligand. The latter hypothesis has been argued previously in the report of altered affinity for ^3^ H]DAMGO in tissue from people who died as a result of suicide [14] as changes in binding affinities are usually due to competition from endogenous ligands [40,41]. More recently, a positron emission tomography (PET) study reported that people with high impulsivity scores released higher levels of endogenous opioids in response to stress [42], resulting in lower ^11^C]carfentanil non-displacable binding potential. Impulsivity has been proposed as a candidate endophenotype for suicidal behaviour [43]; thus, it is possible the decreased OPRM availability in people with schizophrenia who died by suicide is due to increased endogenous opioid levels in response to external stressors. Two such molecules, endomorphin 1 and endomorphin 2, have high affinities and selectivity for OPRM (see [44]) making them prime candidates for future studies.

As with most approaches to the study of the neurobiology of psychiatric disorders, this study has some potentially confounding factors. In particular, we and others [12,13] have shown that ^3^ H]DAMGO binding increases with age. In our study the changes in ^3^ H]DAMGO binding remained when the effect of age was taken into account using an ANCOVA. Another confound is that all people with psychiatric disorders received psychotropic drugs prior to death. Overall, the literature suggests that treatment of rodents with antipsychotics results in either a decrease in the levels of OPRM [45,46] or no change [47] although clozapine, which none of the subjects with schizophrenia received (see Additional file 1: Table S1 for details), has been reported to cause decreases [48]. Evidence against the change in ^3^ H]DAMGO binding in tissue from people with schizophrenia who died by suicide being related simply to the effects of antipsychotic drugs are i) people with schizophrenia who did or did not die by suicide had been treated with antipsychotic drugs, receiving similar levels of final recorded drug doses expressed as chlorpromazine equivalents (Mann Whitney U = 131.0, medians: schizophrenia_non_suicide = 1216, schizophrenia_suicide = 1418;) and the difference in ^3^ H]DAMGO binding is present in all regions between these groups, ii) some of the people with BP had received antipsychotic drugs before death but there was no change in ^3^ H]DAMGO binding in people with this disorder who had died by suicide compared to control subjects and an increase in the BP_non_suicide group.

One potential methodological confound in our study was that the cohort size of people with schizophrenia who died as a result of suicide was relatively small. However, a power analysis ( http://www.biomath.info/power/ttest.htm) showed our study was appropriately powered in BA 9 and 24 (power level 0.8) but was underpowered for the mean differences observed in the CPu (9 additional non-suicide people were needed).

## Conclusions

Our study is the first to suggest there is a decrease in the availability of ^3^ H]DAMGO binding sites in people with schizophrenia who died by suicide. Given the strong link between schizophrenia and the inclination to complete suicide [49], further studies to understand the consequences of changes in the opioid system and its relationship to suicide in schizophrenia are warranted. It is possible that these chemical changes, if detectable by neuroimaging or by a peripheral measure, could be used clinically to identify people with a disposition to suicide which would facilitate the implementation of appropriate interventions [50]. Similarly, our novel, preliminary data on increased ^3^ H]DAMGO binding in BP are intriguing and deserve further investigations. Both findings add to the long held argument that the opioid system plays an important role in the pathophysiology and/or outcomes of psychiatric disorders [51].

## Competing interests

There are no competing financial interests in relation to the work described in this paper. TTM, GP & JN report no competing interests. The following authors have received remuneration in the past: ES received honorarium from Astra-Zeneca and travel support from GSK. BD received travel support from GSK, honorarium from Pfizer, Eli Lilly and MSD.

## Authors’ contributions

ES was involved in the conceptualisation, design, supervision of research and analysis of the project and had the primary responsibility for drafting the manuscript. TTM undertook the radioligand binding and Western blots in the schizophrenia cohort and had input into the manuscript. GP and JN were responsible for the radioligand binding in the suicide cohort and had input into the manuscript. BD was involved in the conceptualisation, design and analysis of the project as well as having significant input into the manuscript. All authors read and approved the final manuscript.

## Pre-publication history

The pre-publication history for this paper can be accessed here:

http://www.biomedcentral.com/1471-244X/12/126/prepub

## Supplementary Material

Additional file 1 **Table S1.** Donor information related to the subjects from whom tissue was used for the measurement of markers for the mu opioid receptor. Description of data: Detailed information of the cohort from whom tissue was sourced for this study, includes demographic data. Click here for file

Additional file 2 **Table S2.** Correlations between demographic and tissue collection data and measures of mu opioid receptors. Description of data: Detailed information of the relationships between potential confounding factors and the experimental measures assessed in this study. Click here for file
