# Supplementary material for: Mu opioid receptor availability in people with psychiatric disorders who died by suicide: a case control study
Source: BMC Psychiatry. 2012 Aug 28;12:126. doi: 10.1186/1471-244X-12-126 (PMC3479023; doi:10.1186/1471-244X-12-126)
Supplement: Additional file 1 — Table S1. Donor information related to the subjects from whom tissue was used for the measurement of markers for the mu opioid receptor. Description of data: Detailed information of the cohort from whom tissue was sourced for this study, includes demographic data. [file 1471-244X-12-126-S1.doc]

Table 1: Donor information related to the subjects from whom tissue was used for the measurement of markers for the mu opioid receptor.

| ID | *Sex* | *Causes of death* | | | | *Anti-cholinergic* | *Suicide* | *Benzodi-azepines* | *Opioids* |
| --- | --- | --- | --- | --- | --- | --- | --- | --- | --- |
| Control | M/F |  |  |  |  | Y/N | Y/N | Y/N |  |
| N = 51 | 34/17 | Crush accident; Thromboembolism; Myocarditis; Exsanguination; IHD; Aortic stenosis; CAA; Acute asthma; Acute myocardial infarction; Cardiomegaly; Acute epiglottitis; Electrocution; Mitral valve prolapse; Ruptured ulcer; Congestive Cardiac Failure; Combined drug toxicity; Cardiomyopathy; Iatrogenic haemorrhage; Pulmonary embolus; Pyelonephritis; Multiple injuries; Pericardial tamponade; Trauma/asphyxia Hypertensive heart disease; Cardiomegaly; Septicaemia; Cardiogenic shock; Haemorrhagic shock; Ruptured infrarenal atheroscletoic aortic aneurysm | | | | 0/51 | 0/51 | 0/51 | Codeine, morphine |
| *Suicide* | *Sex*  M/F | *Causes of death* | | | | *Anti-cholinergic*  Y/N | *Suicide*  Y/N | *Benzodi-azepines*  Y/N | *Opioids* |
| N = 9 | 8/1 | Hanging; Gunshot; CO poisoning | | | | 0/9 | 9/0 | 3/6 | None |
| *Schizophrenia* | *Sex*  M/F | *Causes of death* | *DOI*  *(yrs)* | *Anti-psychotic* | *Chlor Eq.* | *Anti-cholinergic*  Y/N | *Suicide*  Y/N | *Benzodi-azepines*  Y/N | *Opioids* |
| N = 38 | 30/8 | Mediastinitis; CAA;  Multiple injuries;  Bronchopneumonia;  IHD; Cancer;  CO poisoning;  Aspiration;  Hanging; COAD;  Pericarditis;  Meningoencephalitis;  Burning;  Thromboembolism;  Multiple injuries;  Drug overdose;  Intestinal ischaemia;  Pneumonia;  Aspiration; Aneurysm | 19.7 ± 2.5 | Haloperidol; Thioridazine;  Chlorpromazine;  Trifluoperazine;  Pimozide;  Fluphenazine;  Trifluoperazine;  Risperidone;  Flupenthixol; | 1072 ± 99.3 | 19/19 | 12/26 | 14/24 | Morphine  Codeine |
| *MDD* | *Sex*  M/F | *Cause of death* | *DOI*  *(yrs)* | *Anti-depressant* | *Chlor Eq.* | *Other* | *Suicide*  Y/N | *Benzodi-azepines*  Y/N | *Opioids* |
| N = 20 | 10/10 | Pulmonary embolus;  DVT; Combined drug toxicity; CAA; Chest infection; Drug overdose; Hanging; CO poisoning; Drowning; Asphyxia (exhaust) | 13.0 ± 1.9 | Amitriptyline;  Venlafaxine;  Sertraline;  Fluvoxamine; Fluoxetine; Mirtazapine;  Clomipramine;  Nortriptyline;  Citalopram; Mianserin;  Mirtazapine | 382.7 ± 114 | Lithium;  Olanzapine;  Oxycodone;  Zopiclone;  Quetiapine;  Zolpidem;  Risperidone | 15/5 | 6/14 | Methadone |
| *Bipolar Disorder* | *Sex*  M/F | *Cause of death* | *DOI*  *(yrs)* | *Mood Stabiliser* | *Chlor Eq.* | *Other drugs* | *Suicide*  Y/N | *Benzodi-azepines*  Y/N | *Opioids* |
| N = 13 | 8/5 | Combined drug toxicity; Ruptured Aorta; Aspiration; Acute myocardial infarct; Cholecystitis & Cardiomegaly;  IHD; coronary artery disease; Upper airway obstruction; CO poisoning; Hanging; Hypothermia; Drug overdose | 17.0 ± 4.1 | Sodium valproate;  Lithium;  Lamotrigine;  Carbamazepine | 416.9 ± 167 | Fluphenazine  Dothiepin; Benztropine;  Flupenthixol;  Thioridazine;  Droperidol;  Olanzapine;  Zolpidem;  Venlafaxin;  Risperidone;  Chlorpro-mazine;  Paroxetine;  Zuclopen-thizol;  Mogadon | 5/8 | 4/9 | Codeine |

MDD = major depressive disorder; PMI = post-mortem interval; DOI = duration of illness; Chlor Eq. = chlorpromazine equivalents (mg); M/F = Male/Female; Y/N = Yes/No; IHD = ischaemic heart disease; CAA = Coronary artery atheroma; COAD = chronic obstructive airways disease.
