# Supplementary material for: Mu opioid receptor availability in people with psychiatric disorders who died by suicide: a case control study
Source: BMC Psychiatry. 2012 Aug 28;12:126. doi: 10.1186/1471-244X-12-126 (PMC3479023; doi:10.1186/1471-244X-12-126)
Supplement: Additional file 2 — Table S2. Correlations between demographic and tissue collection data and measures of mu opioid receptors. Description of data: Detailed information of the relationships between potential confounding factors and the experimental measures assessed in this study. [file 1471-244X-12-126-S2.docx]

Table S2: Correlations between demographic and tissue collection data and measures of mu opioid receptors.

| Factor | Measure | Group; n | Brain Region | Correlation | F | DF | P value |
| --- | --- | --- | --- | --- | --- | --- | --- |
| Age | [^3^H]DAMGO | **Control; 42** | **BA 24** | **0.3091** | **17.90** | **1, 40** | **0.0001** |
|  |  | Suicide; 9 | BA 24 | 0.1084 | 0.8509 | 1, 7 | 0.3870 |
|  |  | Schizophrenia; 26 | BA 24 | 0.01256 | 0.3053 | 1, 24 | 0.5857 |
|  |  | **Schizophrenia (s); 12** | **BA 24** | **0.3079** | **4.448** | **1, 10** | **0.0611** |
|  |  | **MDD; 5** | **BA 24** | **0.5539** | **3.725** | **1, 3** | **0.1492** |
|  |  | **MDD (s); 15** | **BA 24** | **0.4722** | **11.63** | **1, 13** | **0.0047** |
|  |  | BP; 8 | BA 24 | 0.2877 | 2.423 | 1, 6 | 0.1706 |
|  |  | BP (s); 5 | BA 24 | 0.000007 | 0.0002219 | 1, 3 | 0.9891 |
|  |  |  |  |  |  |  |  |
| PMI | [^3^H]DAMGO | Control; 42 | BA 24 | 0.003495 | 0.1403 | 1, 40 | 0.7100 |
|  |  | Suicide; 9 | BA 24 | 0.03875 | 0.2822 | 1, 7 | 0.6117 |
|  |  | Schizophrenia; 26 | BA 24 | 0.003507 | 0.08447 | 1, 24 | 0.7738 |
|  |  | Schizophrenia (s); 12 | BA 24 | 0.04846 | 0.5092 | 1, 10 | 0.4918 |
|  |  | MDD; 5 | BA 24 | 0.07816 | 0.2544 | 1, 3 | 0.6487 |
|  |  | MDD (s); 15 | BA 24 | 0.007997 | 0.1048 | 1, 13 | 0.7513 |
|  |  | BP; 8 | BA 24 | 0.07319 | 0.4738 | 1, 6 | 0.5169 |
|  |  | BP (s); 5 | BA 24 | 0.09860 | 0.3282 | 1, 3 | 0.6069 |
|  |  |  |  |  |  |  |  |
| pH* | [^3^H]DAMGO | Control; 42* | BA 24 | 0.2171 |  |  | 0.1672 |
|  |  | Suicide; 9* | BA 24 | 0.2667 |  |  | 0.4933 |
|  |  | Schizophrenia; 26 | BA 24 | 0.002189 | 0.05265 | 1, 24 | 0.8205 |
|  |  | Schizophrenia (s); 12 | BA 24 | 0.2912 | 4.109 | 1, 10 | 0.0701 |
|  |  | **MDD; 5** | **BA 24** | **0.6503** | **5.579** | **1, 3** | **0.0992** |
|  |  | MDD (s); 15 | BA 24 | 0.1714 | 2.482 | 1, 12 | 0.1412 |
|  |  | BP; 8 | BA 24 | 0.1020 | 0.6813 | 1, 6 | 0.4407 |
|  |  | BP (s); 5 | BA 24 | 0.1528 | 0.5409 | 1, 3 | 0.5153 |
|  |  |  |  |  |  |  |  |
| Age | [^3^H]DAMGO | **Control; 20** | **BA 24** | **0.3698** | **10.56** | **1, 18** | **0.0044** |
|  |  |  |  |  |  |  |  |
|  |  | **Control; 20** | **BA 9** | **0.3636** | **10.29** | **1, 18** | **0.0049** |
|  |  | Schizophrenia; 26 | BA 9 | 0.2245 | 6.949 | 1, 24 | 0.0145 |
|  |  | **Schizophrenia (s); 12** | **BA 9** | **0.4828** | **9.335** | **1, 10** | **0.0121** |
|  |  |  |  |  |  |  |  |
|  |  | Control; 20 | CPu | 0.02732 | 0.5056 | 1, 18 | 0.4861 |
|  |  | Schizophrenia; 25 | CPu | 0.06902 | 1.631 | 1, 23 | 0.2149 |
|  |  | Schizophrenia (s); 11 | CPu | 0.2194 | 2.530 | 1, 9 | 0.1461 |
|  |  |  |  |  |  |  |  |
| PMI | [^3^H]DAMGO | Control; 20 | BA 24 | 0.03547 | 0.6620 | 1, 18 | 0.4265 |
|  |  |  |  |  |  |  |  |
|  |  | Control; 20 | BA 9 | 0.04585 | 0.8649 | 1, 18 | 0.3647 |
|  |  | Schizophrenia; 26 | BA 9 | 0.04513 | 1.134 | 1, 24 | 0.2975 |
|  |  | Schizophrenia (s); 12 | BA 9 | 0.001586 | 0.01589 | 1, 10 | 0.9022 |
|  |  |  |  |  |  |  |  |
|  |  | Control; 20 | CPu | 0.005341 | 0.09665 | 1, 18 | 0.7595 |
|  |  | Schizophrenia; 25 | CPu | 0.001080 | 0.02378 | 1, 23 | 0.8789 |
|  |  | Schizophrenia (s); 11 | CPu | 0.07695 | 0.7502 | 1, 9 | 0.4089 |
|  |  |  |  |  |  |  |  |
| pH | [^3^H]DAMGO | Control; 20 | BA 24 | 0.07860 | 1.535 | 1, 18 | 0.2312 |
|  |  |  |  |  |  |  |  |
|  |  | Control; 20 | BA 9 | 0.02282 | 0.4203 | 1, 18 | 0.5249 |
|  |  | Schizophrenia; 26 | BA 9 | 0.04003 | 1.001 | 1, 24 | 0.3271 |
|  |  | Schizophrenia (s); 12 | BA 9 | 0.1589 | 1.890 | 1, 10 | 0.1992 |
|  |  |  |  |  |  |  |  |
|  |  | Control; 20 | CPu | 0.1531 | 3.254 | 1, 18 | 0.0880 |
|  |  | Schizophrenia; 25 | CPu | 0.002351 | 0.05184 | 1, 23 | 0.8220 |
|  |  | **Schizophrenia (s); 11** | **CPu** | **0.5476** | **10.90** | **1, 9** | **0.0092** |
|  |  |  |  |  |  |  |  |
| DOI | [^3^H]DAMGO | Schizophrenia; 26 | BA 24 | 0.07490 | 1.943 | 1, 24 | 0.1761 |
|  |  | Schizophrenia (s); 12* | BA 24 | 0.1908 |  |  | 0.5525 |
|  |  |  |  |  |  |  |  |
|  |  | **Schizophrenia; 26** | **BA 9** | **0.2831** | **9.478** | **1, 24** | **0.0051** |
|  |  | Schizophrenia (s); 12* | BA 9 | 0.3887 |  |  | 0.2097 |
|  |  |  |  |  |  |  |  |
|  |  | Schizophrenia; 25 | CPu | 0.1192 | 2.979 | 1, 23 | 0.0984 |
|  |  | Schizophrenia (s); 11* | CPu | -0.1567 |  |  | 0.6454 |
|  |  |  |  |  |  |  |  |
| Chlor. Eq. | [^3^H]DAMGO | **Schizophrenia; 26*** | **BA 24** | **0.4116** |  |  | **0.0570** |
|  |  | Schizophrenia (s); 12 | BA 24 | 0.1236 | 1.410 | 1, 10 | 0.2625 |
|  |  |  |  |  |  |  |  |
|  |  | Schizophrenia; 26* | BA 9 | 0.1621 |  |  | 0.4712 |
|  |  | Schizophrenia (s); 12 | BA 9 | 0.08532 | 0.9328 | 1, 10 | 0.3569 |
|  |  |  |  |  |  |  |  |
|  |  | Schizophrenia; 25 * | CPu | -0.06753 |  |  | 0.7712 |
|  |  | Schizophrenia (s); 11 | CPu | 0.0247 | 0.2279 | 1, 10 | 0.6444 |
|  |  |  |  |  |  |  |  |
| Age | Mu protein | Control; 20 | BA 24 | 0.06872 | 1.328 | 1, 18 | 0.2642 |
|  |  | Schizophrenia; 26 | BA 24 | 0.05586 | 1.420 | 1, 24 | 0.2450 |
|  |  | Schizophrenia (s); 12 | BA 24 | 0.08140 | 0.8861 | 1, 10 | 0.3687 |
|  |  |  |  |  |  |  |  |
|  |  | Control; 20 | BA 9 | 0.2478 | 5.931 | 1, 18 | 0.0255 |
|  |  | Schizophrenia; 26 | BA 9 | 0.1125 | 3.042 | 1, 24 | 0.0939 |
|  |  | **Schizophrenia (s); 12** | **BA 9** | **0.4809** | **9.263** | **1, 10** | **0.0124** |
|  |  |  |  |  |  |  |  |
|  |  | Control; 20 | CPu | 0.01429 | 0.2610 | 1, 18 | 0.6157 |
|  |  | Schizophrenia; 25 | CPu | 0.001531 | 0.03526 | 1, 23 | 0.8527 |
|  |  | Schizophrenia (s); 11 | CPu | 0.0002173 | 0.001956 | 1, 9 | 0.9657 |
|  |  |  |  |  |  |  |  |
| PMI | Mu protein | Control; 20 | BA 24 | 0.06044 | 1.158 | 1, 18 | 0.2961 |
|  |  | Schizophrenia; 26 | BA 24 | 0.006831 | 0.1651 | 1, 24 | 0.6881 |
|  |  | Schizophrenia (s); 12 | BA 24 | 0.01889 | 0.1926 | 1, 10 | 0.6701 |
|  |  |  |  |  |  |  |  |
|  |  | Control; 20 | BA 9 | 0.01158 | 0.2109 | 1, 18 | 0.6515 |
|  |  | Schizophrenia; 26 | BA 9 | 0.08656 | 2.274 | 1, 24 | 0.1446 |
|  |  | Schizophrenia (s); 12 | BA 9 | 5.425e-006 | 5.425e-005 | 1, 10 | 0.9943 |
|  |  |  |  |  |  |  |  |
|  |  | Control; 20 | CPu | 0.1555 | 3.314 | 1, 18 | 0.0854 |
|  |  | Schizophrenia; 25 | CPu | 0.01115 | 0.2594 | 1, 23 | 0.6154 |
|  |  | Schizophrenia (s); 11 | CPu | 0.08412 | 0.8267 | 1, 9 | 0.3869 |
|  |  |  |  |  |  |  |  |
| pH | Mu protein | Control; 20 | BA 24 | 0.005187 | 0.09386 | 1, 18 | 0.7628 |
|  |  | Schizophrenia; 26 | BA 24 | 0.02430 | 0.5976 | 1, 24 | 0.4470 |
|  |  | Schizophrenia (s); 12 | BA 24 | 0.03717 | 0.3861 | 1, 10 | 0.5483 |
|  |  |  |  |  |  |  |  |
|  |  | Control; 20 | BA 9 | 0.1433 | 3.010 | 1, 18 | 0.0998 |
|  |  | Schizophrenia; 26 | BA 9 | 0.08301 | 2.173 | 1, 24 | 0.1535 |
|  |  | **Schizophrenia (s); 12** | **BA 9** | **0.3758** | **6.020** | **1, 10** | **0.0341** |
|  |  |  |  |  |  |  |  |
|  |  | Control; 20 | CPu | 0.02127 | 0.3911 | 1, 18 | 0.5395 |
|  |  | Schizophrenia; 25 | CPu | 6.916e-007 | 1.591e-005 | 1, 23 | 0.9969 |
|  |  | Schizophrenia (s); 11 | CPu | 0.01791 | 0.1642 | 1, 9 | 0.6948 |
|  |  |  |  |  |  |  |  |
| DOI | Mu protein | Schizophrenia; 26 | BA 24 | 0.05630 | 1.432 | 1, 24 | 0.2431 |
|  |  | Schizophrenia (s); 12* | BA 24 | -0.1327 |  |  | 0.6832 |
|  |  |  |  |  |  |  |  |
|  |  | Schizophrenia; 26 | BA 9 | 0.09029 | 2.382 | 1, 24 | 0.1358 |
|  |  | Schizophrenia (s); 12* | BA 9 | -0.2156 |  |  | 0.4990 |
|  |  |  |  |  |  |  |  |
|  |  | Schizophrenia; 25 | CPu | 0.05427 | 1.320 | 1, 23 | 0.2624 |
|  |  | Schizophrenia (s); 11* | CPu | 0.1106 |  |  | 0.7545 |
|  |  |  |  |  |  |  |  |
| Chlor. Eq. | Mu protein | Schizophrenia; 26* | BA 24 | -0.2935 |  |  | 0.1850 |
|  |  | Schizophrenia (s); 12 | BA 24 | 0.02034 | 0.2077 | 1, 10 | 0.6583 |
|  |  |  |  |  |  |  |  |
|  |  | Schizophrenia; 26* | BA 9 | 0.1914 |  |  | 0.3935 |
|  |  | Schizophrenia (s); 12 | BA 9 | 0.0008022 | 0.008028 | 1, 10 | 0.9304 |
|  |  |  |  |  |  |  |  |
|  |  | Schizophrenia; 25 * | CPu | -0.1374 |  |  | 0.5421 |
|  |  | Schizophrenia (s); 11 | CPu | 0.007015 | 0.06358 | 1, 9 | 0.8066 |

* These data were not normally distributed; therefore the relationship between variables was assessed using the Spearman rank correlation test. Factors in bold were considered to have a significant relationship with the experimental variable.
